# Practice characteristics influencing variation in provision of depression care in general practice in Norway; a registry-based cohort study (The Norwegian GP-DEP study)

**DOI:** 10.1186/s12913-022-08579-x

**Published:** 2022-09-26

**Authors:** Sharline Riiser, Valborg Baste, Inger Haukenes, Tone Smith-Sivertsen, Øystein Hetlevik, Sabine Ruths

**Affiliations:** 1 Research Unit for General Practice, NORCE Norwegian Research Centre, Bergen, Norway; 2 Department of Global Public Health and Primary Care, University of Bergen, Bergen, Norway; 3 Division of Psychiatry, Haukeland University Hospital, Bergen, Norway

**Keywords:** Depression, General practice, Drug therapy, Sick leave, Rural health services, Patient list size, Continuity of patient care, Health services research, Large database research

## Abstract

**Background:**

There is growing evidence of variation in treatment for patients with depression, not only across patient characteristics, but also with respect to the organizational and structural framework of general practitioners’ (GPs') practice. However, the reasons for these variations are sparsely examined. This study aimed to investigate associations of practice characteristics with provision of depression care in general practices in Norway.

**Methods:**

A nationwide cohort study of residents aged ≥ 18 years with a new depression episode in general practice during 2009–2015, based on linked registry data. Exposures were characteristics of GP practice: geographical location, practice list size, and duration of GP-patient relationship. Outcomes were talking therapy, antidepressant medication and sick listing provided by GP during 12 months from date of diagnosis. Associations between exposure and outcome were estimated using generalized linear models, adjusted for patients’ age, gender, education and immigrant status, and characteristics of GP practice.

**Results:**

The study population comprised 285 113 patients, mean age 43.5 years, 61.6% women. They were registered with 5 574 GPs. Of the patients, 52.5% received talking therapy, 34.1% antidepressant drugs and 54.1% were sick listed, while 17.3% received none of the above treatments. Patients in rural practices were less likely to receive talking therapy (adjusted relative risk (adj RR) = 0.68; 95% confidence interval (CI) = 0.64–0.73) and more likely to receive antidepressants (adj RR = 1.09; 95% CI = 1.04–1.14) compared to those in urban practices. Patients on short practice lists were more likely to receive medication (adj RR = 1.08; 95% CI = 1.05–1.12) than those on long practice lists. Patients with short GP-patient relationship were more likely to receive talking therapy (adj RR = 1.20; 95% CI = 1.17–1.23) and medication (adj RR = 1.08; 95% CI = 1.04–1.12), and less likely to be sick-listed (RR = 0.88; 95% CI = 0.87–0.89), than patients with long GP-patient relationship.

**Conclusions:**

Provision of GP depression care varied with practice characteristics. Talking therapy was less commonly provided in rural practices and among those with long-lasting GP-patient relationship. These differences may indicate some variation, and therefore, its reasons and clinical consequences need further investigation.

**Supplementary Information:**

The online version contains supplementary material available at 10.1186/s12913-022-08579-x.

## Background

Depression is a leading cause of ill health and contributes greatly to years lived with disability [1], at high personal and societal costs. The Norwegian Institute of Public Health estimated that about 10% of the adult population will have a depressive episode over the course of a year [2]. However, there have been conducted no national diagnosis-based population surveys in Norway, yet.

The World Health Organisation (WHO) highlights the resilience of the primary care sector as an effective response to global challenges, including ageing populations, health inequalities, and increase in mental disorders [3]. Many European health authorities share a common vision of a strong, publicly funded primary health care system, with general practice as a cornerstone [4, 5]. To secure equal access and continuity of primary care, countries like Norway, the Netherlands, Denmark, and the UK have established list-based systems, with each citizen contracted to a specific general practitioner (GP) practice, and the GPs having fixed, personalized patient lists, and acting as gatekeepers to specialist health care [6]. Continuity of GP services is considered an important prerequisite for quality of care [7], and has been shown to improve patient outcomes regarding mental health [8].

In Norway, as in many other European countries, GPs are often the first health care professionals to whom depressed patients present, and thus play a key role in diagnosing and treating depression [4]. According to Norwegian and British national guidelines [9, 10], talking therapy by the GP is the first choice of treatment in mild to moderate depression. In moderate to severe depression, talking therapy combined with antidepressant medication should be considered, and GPs can refer patients to specialised mental care. Patient preferences are crucial regarding treatment decisions. A meta-analytic review across different settings yielded a 70% greater patient preference for psychological treatment over pharmacological treatment for depression [11].

Studies have shown that characteristics of GP practices may influence the provision of care. Urban GP practice location was associated with higher antidepressant prescription levels in France [12] and Scotland [13]. In terms of GP practice list size, the latter study showed lower prescription levels in practices with large list size [13], while a study from East London demonstrated the opposite [14]. However, these studies were conducted among GP practice populations without considering depression diagnoses. In Norway as in the rest of Europe, the impact of factors not directly related to patient characteristics or severity of disease on treatment is poorly documented [15]. Such knowledge may inform policy makers’ decisions on how to improve the organization of health care services.

The aim of this nationwide registry-based study was therefore to investigate associations of practice characteristics with provision of depression care in general practices in Norway.

## Methods

### Setting

In 2013, Norway’s 5.1 million inhabitants lived in 428 municipalities with populations ranging from about 200 to 634 000 people. The municipalities are classified according to Classification of centrality, describing their location in relation to urban settlements of various sizes [16]. Of the municipalities, 16% were urban (the largest cities in Norway), 44% in-between (small towns and larger settlements), and 40% rural (rural and remote rural areas) [17]. Of the Norwegian population, 43% lived in urban municipalities, 44% in in-between municipalities, and 14% in rural municipalities. All inhabitants have equal access to the primary and secondary public health services and prescription drugs (for example antidepressant drugs) covered by the National Insurance Scheme. In 2001, a national list-based system (the Regular GP Scheme) was implemented, giving all residents the right to have a GP, and to change GP twice a year. However, there is limited freedom of choice due to shortage of GPs, especially in rural areas. More than 99% of the population is registered with a specific GP, giving most patients good access to primary mental health care. Although, all GPs in Norway face the same regulations, such as payment systems and constraints, their practice characteristics may vary considerably. GPs are usually required to participate up to one day per week in other clinical or administrative tasks in the municipalities, such as primary care out-of-hours services, preventive health care in school, nursing home and prison. Some GPs are engaged in research and teaching activities, while others work part-time. Treatment in specialist health care by psychologist or psychiatrist requires a referral from the GP and is usually provided to patients with complex conditions, recurrent or severe depression. Access to specialist mental health care is limited and varies across treatment sites, but documentation at the national level shows no variation in waiting lists across urban–rural areas.

### Design

We conducted a nationwide registry-based cohort study comprising all individuals with a new depression diagnosis recorded in general practice in 2009–2015. We examined associations between the characteristics of GP practices and the provision of GP depression care for 12 months from the date of the depression diagnosis.

### Data sources

Information from national registries for the period 2008 through 2016 was linked at the individual patient- and GP-level, using the (encrypted) unique personal identification number assigned to all residents of Norway. Data was stored and analyzed in a safe server at the University of Bergen, Norway.

The source population comprised all inhabitants of Norway born before 1 January 1996 and alive 1 January 2008 (4,017,989 individuals), drawn from the *Population Registry*. We obtained information regarding individuals’ gender, year of birth, immigrant status and degree of urbanity, based on place of residence for all citizens. *The Regular GP Registry* provided information on all participating GPs and their listed patient population. We obtained information on GPs’ list size, i.e., the number of patients registered with the GP. We extracted the date of patients entering their GP’s list, from the start of the Regular GP Scheme in 2001, and eventually the date of patients leaving their GP’s list. *The Control and Reimbursement of Health Care Claims* (KUHR) database stores data on all fee-for-service claims from public primary care providers. For each contact with a GP during daytime, we obtained information on date of contact, diagnoses recorded according to the International Classification of Primary Care, 2^nd^ version (ICPC-2), and reimbursement code(s) for diagnostic and therapeutic measures, as recorded by the GPs. *The Norwegian Prescription Database* (NorPD) contains information on all prescription drugs dispensed to individual patients treated in ambulatory care. For each prescription of an antidepressant drug, we extracted date of dispensing, generic drug information (Anatomical Therapeutic Chemical (ATC) code), and reimbursement code (drugs reimbursed by the Norwegian State for the treatment of depression). *The National Education Database* is based on the International Standard Classification of Education (ISCED 2011). We obtained information on the highest level of completed education.

### Study population

The study population was established by first identifying all individuals aged 18 years or above with a depression diagnosis recorded in general practice (GP consultation with the ICPC-2 code P76 Depression in KUHR) during 01.01.2008–31.12.2015 (n = 443 803). Second, to establish a cohort of patients with a *new* depression diagnosis, we conducted washout of 146 226 patients with a depression diagnosis in general practice (P76 in KUHR) and/or specialist care (ICD-10 codes F32, F33, F34 or F41.2 in NPR) and/or dispensed antidepressant drug (ATC code N06A, referred by the Norwegian State for the treatment for depression in NorPD) during 12-month *prior to index date*. Only patients’ first depression episode during the study period was included (n = 301 577). Finally, we excluded 16 464 patients with missing information on GP practice characteristics or registered on GP patient lists smaller than 300 patients. We did so because these lists contained only a few patients and were probably created administratively or in start-up phase, with no proper functioning. The final study population comprised 285 113 patients, registered with 5574 GP practices.

### Exposure

Exposures studied were characteristics of GP practices; geographical practice location, practice list size and duration of GP-patient relationship. Practice location was categorized as urban (the largest cities in Norway), in-between (small towns and larger settlements), and rural (rural and remote rural areas), with urban practice location as reference. Practice list size was categorized into quintiles (300–848, 849–1075, 1076–1238, 1239–1473, and 1474–2506 patients) based on list length for all GPs in 2012 (in the middle of the study period), with the longest list length as reference. Duration of GP-patient relationship was measured in years, from first date on current GP list until the date of depression diagnosis, categorized into 1–2, 3–4, 5–6, 7–8, 9–14 years, with the longest continuity as reference.

### Outcome

Information on GP depression care linked to ICPC-2 code P76 Depression, provided during 12 months from the index date was identified. Types of GP depression care studied were talking therapy, antidepressant drugs, and certification of sickness absence. Talking therapy was recorded by identifying the reimbursement code for this measure in KUHR. The prerequisites for this reimbursement code were changed during the study period. A referral to secondary mental care was a requirement until July 2011, and until July 2014, this code could not be combined with the higher rewarding reimbursement code for a long consultation (> 20 min). From NorPD we included all dispensed antidepressant drugs (ATC code N06A) reimbursed for the treatment of depression prescribed by GPs and other doctors. In a previous study based on similar datasets, drug treatment for depression was initiated by a GP in 86% of cases [18]. Certification of sickness absence was recorded by identifying the reimbursement code for this measure in KUHR, and was only used for patients aged 18–66 years, because usual age at retirement in Norway is 67 years. All outcome variables were binary (yes, no).

### Covariates

Gender was recorded as men or women. We recoded patient age into six groups: 18–29, 30–39, 40–49, 50–59, 60–69 and 70 + years. Educational level was recoded from 11 levels into three categories: low (primary school, grades 1–7, and lower-secondary school, grades 8–10, or less); medium (upper-secondary school, grades 11–13); and high (> 13 years, university, and higher education). Immigrant status was recoded into Norwegian-born or born abroad of foreign-born parents.

### Statistical analyses

Descriptive statistics were used to examine the distribution of demographic variables in the study population and characteristics of GP practices. The Pearson’s correlation between practice list size and continuity was presented.

The provision of GP depression care during 12 months from date of diagnosis was analyzed as one treatment at the time, because we lack exact knowledge of whether patients receiving more than one treatment option did receive them simultaneously, as overlapping or consecutively. Associations between exposure (practice location, list size and continuity of care) and outcome (talking therapy, antidepressant drugs and sick leave certification) were analyzed with log binomial regression estimating relative risk (RR) with 95% CI. Due to problems with converge in a log binomial regression regarding talking therapy, Poisson regression with robust variance estimates (Zou, 2004), was applied to estimate RR and 95% CI. Crude estimates and estimates adjusted for patient characteristics and GP practice characteristics were presented for talking therapy, antidepressant drug and sick leave certification. In the regression analyses, age is used as a continuous adjustment variable. We decided to present list size and duration of GP-patient relationship in categories because it was easier to interpret the results and it was not obvious that there would be a linear relationship with the outcomes. However, when list size and duration were used as adjustment variables, they were used as continues variables. Since age can be a confounding factor for the association between duration of GP-relationship and talking therapy, age stratified corresponding models were applied in sensitivity analyses. For all statistical analyses, we used α = 0.05 as significant level. The data were analyzed by using STATA/SE version 16.1 (Stata Statistical Software).

## Results

The study population comprised 285 113 patients (61,6% women) with a new depression episode in general practice, mean age 43.5 (SD = 16.3) years; the youngest age group (18–29 years) constituted the largest group with 24.2% of the cohort, and the oldest age group (70+) constituted the smallest group with 7.2% (Table 1). Educational attainment was high among 27.0% of the patients, medium among 41.2% and low among 31.8%. 11,7% were born abroad of foreign-born parents.

**Table 1:** Characteristics of 285 113 patients with a new depression diagnosis in general practice in Norway, 2009–2015

	n	%
**Gender**		
Women	175 553	61.6
Men	109 560	38.4
**Age group, years**		
18–29	69 095	24.2
30–39	61 814	21.7
40–49	61 869	21.7
50–59	46 421	16.3
60–69	25 234	8.9
70 +	20 590	7.2
**Educational level**^a^		
Low	89 231	31.8
Medium	115 827	41.2
High	75 947	27.0
**Immigrant status**		
Born abroad	33 227	11.7
Norwegian-born, other groups	251 886	88.3

Percentages are based on valid numbers. Missing data on educational level, *n* = 4108

^a^ Educational level: Low = primary school (grades 1–7) and lower secondary school (grades 8–10), or less; Medium = upper-secondary school (grades 11–13); High = university and higher education

The patients in the study population were registered on 5 574 unique GP patient lists. GP practice location was distributed as urban (37.9%), in-between (45.5%), or rural (16.6%). GPs’ mean list size was 1 328 (SD = 379) patients and decreased with decreasing degree of urbanity (Table 2). The mean duration of the GP-patient relationship was 70 (SD = 50) months and was slightly shorter among patients in rural practices. Duration of GP-patient relationship increased with increasing list size (correlation coefficient = 0.29, *p* < 0.000).

**Table 2:** Characteristics of 5574 GP practices in Norway, per index year, 2009–2015

	GP practices N (%)	List size, number of patients Mean (SD)	Duration of GP-patient relationship, months Mean (SD)
**All GP practices**	5574 (100%)	1328 (379)	70 (50)
**GP practice location**			
Urban	2 113 (37.9)	1433 (364)	70 (50)
In-between	2 538 (45.5)	1272 (361)	71 (50)
Rural	923 (16.6)	981 (297)	67 (50)

*GP* General practitioner

*SD* Standard deviation

Thirty-six percent has had the same GP for 9–14 years at date of depression diagnosis. The proportions among patients 40 years and older were 42% (40–49 years) and 49% (70 + years). Conversely, 27% of the patients younger than 40 years has had the same GP for only 1–2 years, with higher proportions in age groups 18–29 years (38%) and 30–39 years (28.9%) (not tabulated).

Of the patients, 52.5% received talking therapy, 34.1% antidepressant drugs, 54.1% were sick listed, and 17.3% received only GP consultation with none of the above treatment options. 42.3% of them received more than one treatment option during the 12-month follow-up period (Table 3).

**Table 3:** GP depression care provided to 285 113 patients with a new depression diagnosis in 2009–2015, during 12 months from date of diagnosis

	Patients who received GP depression care	
GP depression care option^a^	n	%
Talking therapy and sick leave certification^b^	48 381	17.0
Sick leave certification only^2^	48 157	16.9
Talking therapy only	42 233	14.8
Talking therapy and antidepressant drug and sick leave certification^b^	30 912	10.8
Talking therapy and antidepressant drug	28 064	9.8
Antidepressant drug only	24 813	8.7
Antidepressant drug and sick leave certification^b^	13 352	4.7
GP consultation for depression with none of the options above	49 201	17.3
Total	285 113	100

^a^ Talking therapy, antidepressant drug, sick leave certification

^b^ Sick leave certification applies for patients aged 20–66 years only, because usual age for retirement in Norway is 67 years

In terms of treatment rates (percentage of patients receiving a treatment), talking therapy was more commonly provided to younger compared to older patients, to those with high compared to low educational level, and to immigrants compared to Norwegian-born. Regarding drug treatment, patients being male, older, low or medium educated, or immigrant, were more likely to receive antidepressants compared to their counterparts. Sick leave certification was more commonly provided to patients being female or highly educated (Table 4).

**Table 4:** GP depression care^1^ provided to 285 113 patients with a new depression diagnosis (2009–2015) during 12 months from date of diagnosis, by patient characteristics

		**GP depression care options**^a^							
		**Talking therapy**			**Antidepressant drug**			**Sick leave certification**^**b**^	
	**Number of patients**	%	Mean n		%	Mean n		%	Mean n
**Gender**									
Women	175 533	52.5	2.8		33.2	3.7		55.0	3.8
Men	109 560	51.9	2.8		35.5	3.6		53.5	3.8
**Age group, years**									
18–29	69 095	56.7	2.6		33.2	3.3		46.6	3.4
30–39	61 814	56.1	3.0		31.0	3.4		61.8	4.0
40–49	61 869	53.4	3.0		31.7	3.5		60.1	4.0
50–59	46 421	49.9	2.9		32.2	3.6		53.8	3.8
60–69	25 234	45.8	2.6		36.4	3.8		40.9	3.8
70 +	20 590	38.6	2.2		54.5	4.8			
**Educational Level**^**c**^									
Low	89 231	51.7	2.7		36.5	3.6		47.3	3.7
Medium	115 827	51.0	2.8		34.6	3.7		56.2	3.8
High	75 947	55.0	3.0		30.2	3.7		59.8	3.9
**Immigrant status**									
Born abroad	33 227	54.2	2.9		35.8	2.9		53.2	3.7
Norwegian-born, other groups	251 882	52.2	2.8		33.8	3.7		54.5	3.8

Percentages are based on valid numbers. Missing data on educational level, *n* = 4108

^a^ Patients may have received more than one treatment option

^b^ Only patients aged 20–66 years (*n* = 257 645), because usual age for retirement in Norway is 67 years

^c^ Educational level: Low = primary school (grades 1–7) and lower secondary school (grades 8–10) or less; Medium = upper-secondary school (grades 11–13);

High = university and higher education

**Fig. 1:**
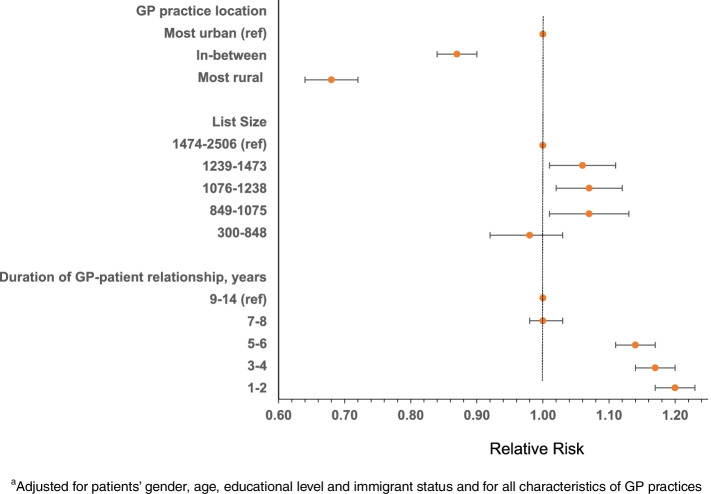
^a^Adjusted RR with 95% CI of receiving talking therapy from GP, among patients with a new depression diagnosis in 2009–2015, by characteristics of GP practices

Patients living outside the largest cities in Norway were less likely to receive talking therapy when consulting their GP with a new depression episode (Fig. 1, Supplementary Table 1). This was particularly evident in the most rural parts of Norway [adj RR = 0.68 (0.64–0.72)]. We found a higher likelihood of receiving talking therapy among patients registered with GPs with medium list sizes compared to those on the longest lists. Regarding continuity of care, patients with a relationship to their GP lasting up to 6 years had a higher likelihood of receiving talking therapy compared with those having a long relationship (9–14 years). Adjusted RR for those with the shortest relationship was = 1.20 (1.17–1.23). Adjusting for patient characteristics and other GP list characteristics had limited impact on the likelihood of receiving talking therapy. Age stratified analyses gave similar results for both strata, 18–39 and 40 + years.

**Fig. 2:**
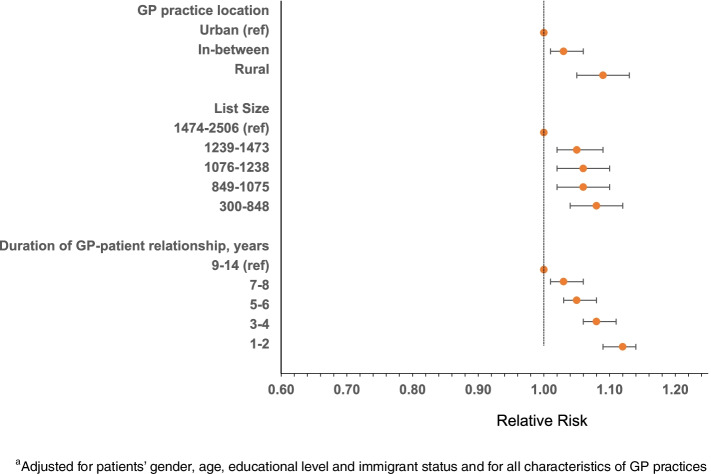
^a^Adjusted RR with 95% CI of receiving antidepressant medication from GP among patients with a new depression diagnosis in 2009–2015, by characteristics of GP practices

Rural practice was associated with a greater likelihood of being prescribed antidepressant drugs [adj RR = 1.09 (1.05–1.13)] compared to urban practice (Fig. 2, Supplementary Table 2). Further, shorter GP list sizes compared to longest list size, and shorter GP-patient relationship compared to longest relationship was associated with a greater likelihood of being prescribed antidepressant drugs.

**Fig. 3:**
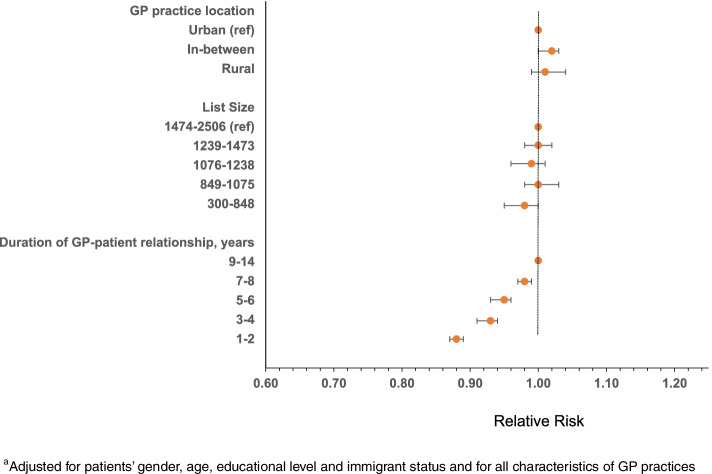
^a^Adjusted RR with 95% CI of receiving sick leave certification from GP (yes/no), among patients aged 18–66 years with a new depression diagnosis in 2009–2015, by characteristics of GP practices

Among patients at working age, less sick leave certification was associated with shorter GP-patient relationship. Adj RR for those with the shortest relationship (1–2 years) was = 0.88 (0.87–0.89) compared to longest relationship (9–14 years) (Fig. 3, Supplementary Table 3).

## Discussion

### Summary

In a nationwide cohort of patients with a new depression diagnosis in general practice in 2009–2015, we examined associations between GP practice characteristics and provision of GP depression care. Rural practice location was associated with less provision of talking therapy and more prescription of antidepressant drugs than urban practice location. Short patient lists slightly increased the likelihood of receiving medication compared to long patient lists. Short GP-patient relationship was associated with more talking therapy and antidepressant drugs, and less sick leave certification compared to long GP-patient relationship.

### Strengths and limitations

The main strength of this study is the use of national registry data linked at the individual patient and GP level, providing a rich source of information, and eliminating recall bias. The almost complete data sets are another strength; information only regarding educational level was lacking for 1.4% (*n* = 4108) of the patients, with no expected impact on the results due to small numbers.

GP-registered new depression diagnosis was defined as a GP consultation with the ICPC-2 code P76, after a one-year washout period. The study population was restricted to patients whose depression was identified by a GP and recorded as such. Patients with depression who did not seek help or were not diagnosed with depression by the GP remained beyond the scope. Further, we lack information regarding severity because the ICPC-2 system does not allow for such grading. Consequently, we were not able to examine GPs’ guideline adherence. However, there is no reason to assume that severity of depression is unevenly distributed across the exposure variables. A limitation is that we study reimbursement codes recorded for payment purposes, not a report on what the GPs actually did. The prerequisites for the reimbursement code for talking therapy were changed during the study period. Further, this reimbursement code must be registered manually by the GP, in contrast to the reimbursement code for sick leave certification, which is registered automatically. Although we may have underestimated the prevalence of performed GP talking therapy sessions during the first years of the study period, this would apply for all GPs and cause “non-differential” misclassification. The NorPD contains complete data on prescription drugs dispensed. Although we may have slightly underestimated ‘prescribed’ antidepressants due to primary non-compliance, the use of drug dispensing data is recognized as an acceptable proxy for drug use in epidemiological studies [19]. Another limitation of the Norwegian registries is lack of other information that might explain practice variation, e.g., GPs’ experience and fields of interest, full-time-equivalent working as GP, treatment in specialist health care and/or municipal psychiatric services and waiting lists.

We measured continuity of care as the period from a person was registered on a given GP list to the date of depression diagnosis. Registration of GP-patient relationships in the RGP registry began with the establishment of the RGP scheme in 2001. This means that the study population, regardless of age at date of diagnosis, may have achieved maximum continuity between 9 years (index year 2009) and 14 years (index year 2015). The real continuity may be even longer because many patients chose the GP they previously went to (the "municipal doctor") as their “regular GP” in 2001; however, the possible implications on the study outcomes could not be examined due to lack of data.

We believe that the results of this study that started in 2009 are still valuable, because there haven’t been any healthcare reforms that may have impacted general practice care during this time frame. Whether the results of this study, shedding light on how practice characteristics affect service delivery in Norway, may be transferable to countries with similar organization of primary care and mental healthcare provision, such as UK, Sweden, Denmark, and the Netherlands remains to be clarified.

### Interpretation of findings and comparison with existing literature

#### Geographical practice location

In the present study we found that rural practice location was associated with less provision of talking therapy and somewhat higher prescription of antidepressant drugs compared with urban practice location. These results are not in line with studies from general practice in Scotland [13] and France [12] which found lower levels of antidepressant prescribing for rural practices. However, the Scottish and French studies did not select GP practice populations on basis of depression diagnoses. Several factors may influence the geographical variation in drug prescribing. GP practices in rural areas compared to cities have a higher turnover of GPs [20], and relatively more patient lists are served by substitute/temporary doctors, who may be less experienced or competent in offering talking therapy. Another explanation could be a higher workload in rural practices [21] due to a general shortage of GPs in rural areas, and therefore less opportunity to offer follow-up with talking therapy sessions. Long distance to the GP practice may also contribute to less talking therapy, to the benefit of prescriptions of medication that is readily available. Considering rural populations, studies have found a high rate of barriers related to stigma, which may affect help-seeking behavior more in rural than urban residing patients [22, 23]. Patients postponing the visit to the doctor may become sicker and have a greater need for medication. Differences in behavior and demand between patients in urban and rural areas, as well as the doctors’ attitude, may therefore affect treatment decisions. Nevertheless, the question arises as to whether patients in rural areas in general have less access to talking therapy, reflecting inequity in depression treatment. This is concerning because guidelines emphasize psychological treatment as GPs’ primary tool in depression care [9]. Notably, a meta-analytic review across different settings yielded a 70% greater patient preference for psychological treatment over pharmacological treatment for depression [11].

#### Practice list size

Surprisingly, there was a slightly greater likelihood of receiving antidepressant treatment among patients on short GP lists, which contradicts the idea of a “high-prescriber” profile due to a greater workload among GPs with long patient lists. Our findings align with two studies from general practice in London [14] and Scotland [13]. However, these studies defined list sizes per full-time equivalent GP, in contrast to our study describing actual list size per GP. Regarding talking therapy, we found a slightly greater likelihood of receiving this treatment option with list size between 849–1473 patients compared to the longest list size, possibly explained by GPs with shorter lists have more time to offer talking therapy. We consider list size an appropriate proxy for GPs’ availability for their patients. In 2015, a list size of 300 patients corresponded to approximately one GP practice day a week. This may possibly explain why the shortest patient lists (300–848) were not associated with more talking therapy.

#### Continuity of care

Patients with a short GP-relationship had a higher chance of receiving talking therapy. At a national level, Norwegian GPs have increased the provision of talking therapy to patients with new depression over the last decade [21]. This trend is in accordance with recommendations from Norwegian and international guidelines [9, 10]. Nevertheless, findings from the current study give nuances to this picture by pointing to some variation in talking therapy by continuity of GP-patient relationship that is independent of patient characteristics. Regarding antidepressant treatment, patients with the shortest GP-patient relationship had a relatively higher chance of receiving medication. There are plausible explanations for these variations. A long GP-patient relationship requires physicians who remain in their GP practices over time and thereby have an accumulated knowledge and experience with their list patients, and their diseases and individual needs. Possibly, GPs with short GP-patient relationships are newly qualified doctors who are not yet familiar to their patients and, therefore, may be more willing to initiate medication and talking therapy than GPs with longer GP-patient relationships who possibly practice “wait-and-see” more commonly. GPs may not want to jeopardize the GP-patient relationship and be complaisant to patients’ request for a certain treatment, for instance antidepressant medication. However, this probably applies for all GPs and should not contribute to variation across continuity of care. Long GP-patient relationship (9–14 years) was more common among patients 40 years and older, while short GP-patient relationship (1–2 years) was more common among those younger than 40 years. It has been documented that referral rates to specialist health care among older patients in Norway and the UK were lower than for younger patients [24, 25]. Our analysis was adjusted for patients’ age, we also performed stratified analyzes by age, which gave similar estimates in both strata. Although continuity of care is regarded as an aspect of quality, our findings challenge the idea of long continuity being exclusively good for the patients. Long GP-patient relationship can possibly lead to doctors being less engaged to the patients, or, on the contrary, having a long GP-patient relationship and knowing your patients background may be a starting point for supportive talks instead of formalized talking therapy, and can possibly be therapeutic in itself. However, the findings still implicate inequality of opportunity to receive talking therapy across continuity of GP-patient relationship. Our findings as regards to lesser sick leave certification with short GP-patient relationship may possibly be explained by younger doctors being more restrictive with sick leave certification as national guidelines urges GPs to limit sick leave certification.

## Conclusion

The Norwegian RGP scheme is considered a successful public service [26], with equitable health services for all inhabitants as a core value. Our findings point at some variation due to organizational and structural factors for patients with a new depression episode presented in general practice. Especially, differential treatment applies to talking therapy being less commonly provided in rural practices, and with long-lasting GP-patient relationship. Potentially poorer access to this core treatment modality may be relevant for the population group affected. Therefore, it should be of particular concern to health authorities and decision-makers, as a goal of securing the quality and equality of care regardless of practice characteristics. Although, the differences found in the present study are relatively small, they are relevant at a population level, given a national health policy aiming to provide equity in health care. Further studies are needed to explore the reasons for the observed differences and the implications for patient outcomes.

## Supplementary Information

**Additional file 1: Supplementary Table 1.** Likelihood^a^ (crude and adjusted) of receiving talking therapy from GP (yes/no), among 285 113 patients with a new depression diagnosis in 2009-2015, by characteristics of GP practices. **Supplementary Table 2.** Likelihood^a^ (crude and adjusted) of receiving antidepressant medication from GP (yes/no) among 285 113 patients with a new depression diagnosis in 2009-2015, by characteristics of GP practices. **Supplementary Table 3.** Likelihood^a^ (crude and adjusted) of receiving sick leave certification from GP (yes/no), among 257 645^b^ patients at working age with a new depression diagnosis in 2009-2015, by characteristics of GP practices.

## Data Availability

The data used in this study are provided by Statistics Norway, the Norwegian Directorate of Health, and the Norwegian Institute of Public Health, with restrictions only to be used under licence for researchers in the current study, and so are not publicly available. However, the registry data used in this study will be available from the authors upon reasonable request and with included permission from Regional Ethical Committee for Medical and Health Research Ethics, Region West, Norwegian Data Protection Authority, Statistics Norway, the Norwegian Directorate of Health, and the Norwegian Institute of Public Health.

## Abbreviations

ATC: Anatomical therapeutic chemical
CI: Confidence interval
GLM: Generalized linear model
GP: General practitioner
ICD: International classification of diseases
ICPC: International classification of primary care
KUHR: Control and reimbursement of primary health care claims database
NorPD: Norwegian prescription database
NPR: Norwegian patient registry
RR: Relative risk
SD: Standard deviation
WHO: World health organisation
