# Supplementary material for: Practice characteristics influencing variation in provision of depression care in general practice in Norway; a registry-based cohort study (The Norwegian GP-DEP study)
Source: BMC Health Serv Res. 2022 Sep 26;22:1201. doi: 10.1186/s12913-022-08579-x (PMC9511786; doi:10.1186/s12913-022-08579-x)
Supplement: Supplementary file 1 — Additional file 1: Supplementary Table 1. Likelihooda (crude and adjusted) of receiving talking therapy from GP (yes/no), among 285 113 patients with a new depression diagnosis in 2009-2015, by characteristics of GP practices. Supplementary Table 2. Likelihooda (crude and adjusted) of receiving antidepressant medication from GP (yes/no) among 285 113 patients with a new depression diagnosis in 2009-2015, by characteristics of GP practices. Supplementary Table 3. Likelihooda (crude and adjusted) of receiving sick leave certification from GP (yes/no), among 257 645b patients at working age with a new depression diagnosis in 2009-2015, by characteristics of GP practices. [file 12913_2022_8579_MOESM1_ESM.docx]

| **Supplementary Table 1** Likelihood^a^ (crude and adjusted) of receiving talking therapy from GP (yes/no), among 285 113 patients with a new depression diagnosis in 2009-2015, by characteristics of GP practices | | | | | | | | | |
| --- | --- | --- | --- | --- | --- | --- | --- | --- | --- |
|  | | | **Talking therapy** | | | | | | |
|  | **Patients** |  | **Yes** |  | **Crude** | |  | **Adjusted^b^** | |
| **Characteristics of GP practices** | **n** |  | **n (%)** |  | **RR** | **95% CI** |  | **RR** | **95% CI** |
| **GP Practice location** |  |  |  |  |  |  |  |  |  |
| Urban | 138 883 |  | 79 149 (57.0) |  | 1 |  |  | 1 |  |
| In-between | 124 727 |  | 62 009 (49.7) |  | **0.87** | **0.84-0.90** |  | **0.87** | **0.84-0.90** |
| Rural | 21 503 |  | 8 432 (39.2) |  | **0.69** | **0.65-0.73** |  | **0.68** | **0.64-0.72** |
|  |  |  |  |  |  |  |  |  |  |
| **List size: number of patients**  **on GP list (quintiles)** |  |  |  |  |  |  |  |  |  |
| 1474-2506 | 96 134 |  | 49 917 (51.9) |  | 1 |  |  | 1 |  |
| 1239-1473 | 65 649 |  | 35 493 (54.1) |  | 1.04 | 0.99-1.09 |  | **1.06** | **1.01-1.11** |
| 1076-1238 | 55 505 |  | 29 938 (53.9) |  | 1.04 | 0.99-1.09 |  | **1.07** | **1.02-1.12** |
| 849-1075 | 42 818 |  | 22 289 (52.1) |  | 1.00 | 0.95-1.05 |  | **1.07** | **1.01-1.13** |
| 300-848 | 25 007 |  | 11 953 (47.8) |  | **0.92** | **0.87-0.97** |  | 0.98 | 0.92-1.03 |
|  |  |  |  |  |  |  |  |  |  |
| **Duration of GP-patient**  **relationship, years** |  |  |  |  |  |  |  |  |  |
| 9-14 | 103 234 |  | 48 318 (46.8) |  | 1 |  |  | 1 |  |
| 7-8 | 34 013 |  | 16 388 (48.2) |  | **1.03** | **1.00-1.05** |  | 1.00 | 0.98-1.03 |
| 5-6 | 30 850 |  | 16 936 (54.9) |  | **1.17** | **1.14-1.21** |  | **1.14** | **1.11-1.17** |
| 3-4 | 40 876 |  | 23 159 (56.7) |  | **1.21** | **1.18-1.24** |  | **1.17** | **1.14-1.20** |
| 1-2 | 76 140 |  | 44 789 (58.8) |  | **1.26** | **1.22-1.29** |  | **1.20** | **1.17-1.23** |

**^a^** Results from negative binomial regression estimating relative risk (RR) with 95% confidence interval (CI)

**^b^** Adjusted for patients’ gender, age, educational level and immigrant status and for all characteristics of GP practices

| **Supplementary Table 2** Likelihood^a^ (crude and adjusted) of receiving antidepressant medication from GP (yes/no) among 285 113 patients with a new depression diagnosis in 2009-2015, by characteristics of GP practices | | | | | | | | | |
| --- | --- | --- | --- | --- | --- | --- | --- | --- | --- |
|  |  |  | **Antidepressant medication** | | | | | | |
|  |  |  |  |  | **Crude** | |  | **Adjusted^b^** | |
| **Characteristics of GP practices** | **Patients** |  | **Yes** |  | **RR** | **95% CI** |  | **RR** | **95% CI** |
|  | **n** |  | **n (%)** |  |  |  |  |  |  |
| **GP Practice location** |  |  |  |  |  |  |  |  |  |
| Urban | 138 883 |  | 45 386 (32.7) |  | 1 |  |  | 1 |  |
| In-between | 124 727 |  | 41 431 (34.8) |  | **1.07** | **1.04-1.09** |  | **1.03** | **1.03-1.06** |
| Rural | 21 503 |  | 8 324 (38.7) |  | **1.18** | **1.14-1.23** |  | **1.09** | **1.05-1.13** |
|  |  |  |  |  |  |  |  |  |  |
| **List size: number of patients**  **on GP list (quintiles)** |  |  |  |  |  |  |  |  |  |
| 1474-2506 | 96 134 |  | 30 810 (32.1) |  | 1 |  |  | 1 |  |
| 1239-1473 | 65 649 |  | 22 238 (33.9) |  | **1.06** | **1.02-1.10** |  | **1.05** | **1.01-1.08** |
| 1076-1238 | 55 505 |  | 19 400 (34.9) |  | **1.09** | **1.05-1.13** |  | **1.06** | **1.02-1.10** |
| 849-1075 | 42 818 |  | 15 290 (35.7) |  | **1.11** | **1.08-1.15** |  | **1.06** | **1.02-1.10** |
| 300-848 | 25 007 |  | 9 358 (37.4) |  | **1.17** | **1.13-1.21** |  | **1.08** | **1.04.1.12** |
|  |  |  |  |  |  |  |  |  |  |
| **Duration of GP-patient**  **relationship, years** |  |  |  |  |  |  |  |  |  |
| 9-14 | 103 234 |  | 34 183 (33.1) |  | 1 |  |  | 1 |  |
| 7-8 | 34 013 |  | 11 269 (33.1) |  | 1.00 | 0.98-1.02 |  | **1.03** | **1.01-1.05** |
| 5-6 | 30 850 |  | 10 309 (33.4) |  | 1.01 | 0.98-1.03 |  | **1.05** | **1.02-1.07** |
| 3-4 | 40 876 |  | 14 153 (34.6) |  | **1.05** | **1.02-1.07** |  | **1.08** | **1.05-1.10** |
| 1-2 | 76 140 |  | 27 227 (35.8) |  | **1.08** | **1.06-1.10** |  | **1.12** | **1.10-1.15** |

**^a^** Results from generalized linear model estimating relative risk (RR) with 95% confidence interval (CI)

**^b^** Adjusted for patients’ gender, age, educational level and immigrant status, and for all characteristics of GP practices

| **Supplementary Table 3** Likelihood^a^ (crude and adjusted) of receiving sick leave certification from GP (yes/no), among 257 645^b^ patients at working age with a new depression diagnosis in 2009-2015, by characteristics of GP practices | | | | | | | | | | |
| --- | --- | --- | --- | --- | --- | --- | --- | --- | --- | --- |
|  |  |  | **Sick leave certification** | | | | | | |  |
|  |  |  |  |  | **Crude** | |  | **Adjusted^c^** | |  |
| **Characteristics of GP practices** | **Patients** |  | **Yes** |  | **RR** | **95% CI** |  | **RR** | **95% CI** |  |
|  | **n** |  | **n (%)** |  |  |  |  |  |  |  |
| **GP Practice location** |  |  |  |  |  |  |  |  |  |  |
| Urban | 126 959 |  | 68 936 (54.3) |  | 1 |  |  | 1 |  |  |
| In-between | 112 335 |  | 61 342 (54.6) |  | 1.01 | 0.99-1.02 |  | **1.02** | **1.01-1.04** |  |
| Rural | 18 451 |  | 9 826 (53.2) |  | 0.97 | 0.96-1.01 |  | 1.01 | 0.98-1.03 |  |
|  |  |  |  |  |  |  |  |  |  |  |
| **List size: number of patients**  **on GP list (quintiles)** |  |  |  |  |  |  |  |  |  |  |
| 1474-2506 | 97 459 |  | 48 151 (55.1) |  | 1 |  |  | 1 |  |  |
| 1239-1473 | 59 375 |  | 32 529 (54.8) |  | 1.04 | 0.99-1.09 |  | 1.00 | 0.98-1.02 |  |
| 1076-1238 | 49 950 |  | 26 957 (54.0) |  | 1.04 | 0.99-1.09 |  | 0.99 | 0.97-1.02 |  |
| 849-1075 | 38 376 |  | 20 689 (53.9) |  | 1.00 | 0.95-1.05 |  | 1.00 | 0.97-1.02 |  |
| 300-848 | 22 503 |  | 11 777 (52.3) |  | **0.92** | **0.87-0.97** |  | 0.98 | 0.96-1.01 |  |
|  |  |  |  |  |  |  |  |  |  |  |
| **Duration of GP-patient**  **relationship, years** |  |  |  |  |  |  |  |  |  |  |
| 9-14 | 89 775 |  | 51 624 (57.5) |  | 1 |  |  | 1 |  |  |
| 7-8 | 31 200 |  | 17 493 (56.1) |  | 1.00 | 0.97-1.02 |  | **0.98** | **0.97-0.99** |  |
| 5-6 | 28 509 |  | 15 473 (54.3) |  | 0.98 | 0.96-1.00 |  | **0.95** | **0.94-0.97** |  |
| 3-4 | 37 592 |  | 20 199 (53.7) |  | 0.98 | 0.95-1.00 |  | **0.93** | **0.92-0.95** |  |
| 1-2 | 70 569 |  | 35 314 (50.0) |  | **0.95** | **0.93-0.97** |  | **0.88** | **0.87-0.89** |  |

^a^ Results from generalized linear model estimating relative risk (RR) with 95% confidence interval (CI)

^b^ Only patients aged 18-66 years, because usual age for retirement in Norway is 67 years

**^c^** Adjusted for patients’ gender, age, educational level, and immigrant status, and for all characteristics of GP practices
